# New Potential of Roxatidine Acetate Hydrochloride on Atopic Dermatitis Mouse Model, Human Keratinocytes, and Human Skin Equivalent Model

**DOI:** 10.3389/fphar.2021.797086

**Published:** 2021-12-24

**Authors:** Yun-Mi Kang, Minho Lee, Hyo-Jin An

**Affiliations:** ^1^ Department of Pharmacology, College of Korean Medicine, Sangji University, Wonju, South Korea; ^2^ Department of Life Science, Dongguk University, Seoul, South Korea

**Keywords:** atopic dermatitis, roxatidine acetate chloride, aryl hydrocarbon receptor, sirtuin1, nuclear factor kappa B

## Abstract

Atopic dermatitis (AD) is a complex inflammatory skin disorder, characterized by a complicated pathophysiology and a wide range of clinical phenotypes. Roxatidine acetate chloride (RXA) is a precursor of Roxatidine and a histamine H_2_ receptor antagonist, used for the treatment of gastric ulcers. In this study, we aimed to examine whether RXA had anti-AD effects and determine the underlying molecular mechanism of RXA. The anti-AD effects were examined in *Dermatophagoides farinae* body (Dfb)-induced AD mouse model, tumor necrosis factor (TNF)-α/interferon (IFN)-γ-stimulated HaCaT keratinocytes, and human skin equivalent model using ELISA, histological analysis, immunohistochemistry, Western blot, and immunofluorescence. Results showed that RXA treatment significantly alleviated Dfb-induced AD skin symptoms and clinical severity in mice by decreasing the levels of immunoglobulin E, histamine, and inflammatory cytokines. RXA effectively inhibited the expression of adhesive molecules and recovered the filaggrin expression in Dfb-induced AD skin lesions and TNF-α/IFN-γ-stimulated HaCaT keratinocytes. Additionally, RXA significantly upregulated the expression of aryl hydrocarbon receptor and sirtuin1. The anti-AD effects of RXA were associated with suppressed nuclear factor kappa cascade. Overall, our results suggest that RXA may be a potential anti-AD candidate owing to its inhibitory effect against skin inflammation and protection of the skin barrier function in AD.

## Introduction

Roxatidine acetate hydrochloride (2-acetoxy-N-[3-[m-(1-piperidinylmethyl) phenoxy] propyl] acetamide hydrochloride, RXA) is a competitive histamine H_2_ receptor antagonist, that upon oral absorption, is rapidly converted to its active metabolite, Roxatidine. By competitively inhibiting the binding of histamine to H_2_ receptors, Roxatidine reduces both intracellular cyclic AMP concentrations and gastric acid secretion by parietal cells (Murdoch and McTavish, 1991). Thus, it has been used clinically such as Roxit®, Roxan®, Rozaltat® for an anti-ulcer agent (Ichikawa et al., 1999). Because Roxatidine is relatively safe medicine and absorbed almost (greater than 95%) and quickly after oral administration, it can be applied to other symptoms. So, we tried to investigate about the other biological effects of Roxatidine. In addition, as one of the results among our trials, we have previously reported the anti-inflammatory activities of Roxatidine and inhibition of NF-κB and p38 MAPK activation in LPS-induced RAW 264.7 macrophages (Cho et al., 2011), and the inhibitory effect of Roxatidine on mast cell-mediated allergic response *via* suppression of NF-κB and p38 MAPK (M. Lee et al., 2017). In continuation with our previous study that reported the anti-inflammatory and anti-allergic effect of RXA, we postulated that RXA could also play a role in skin inflammatory disease. In addition, since our previous results demonstrated that RXA could remarkably suppress the level of histamine in PMACI-stimulated human mast cell, we hypothesized that it may also play a key role in the pathogenesis of atopic dermatitis (AD).

AD is the most common chronic inflammatory skin disease, with a high prevalence of up to 20% (Weidinger and Novak, 2016). The primary pathogenic role of skin barrier abnormalities in AD have been highlighted in both patients and experimental models (Elias and Schmuth, 2009). The skin acts as the first line of defense by creating a mechanical and immune barrier. It protects against water loss from the internal to the external environment and protects the individual from external injuries, such as ultraviolet radiation, microorganisms, and allergens. The outermost layers of the epidermis consist of multiple components which are important for maintaining the skin barrier function, including proteins such as filaggrin, proteins that form tight junction, and components of the innate immune system (Hogan et al., 2012). In particular, loss-of function mutations in the gene encoding filaggrin are strongly associated with the development of AD. The deficiency of filaggrin in AD patients contribute to an impaired skin barrier function of the epidermis, which subsequently increases trans-epidermal water loss, pH alterations, and the risk for microbial infection or development of other atopic diseases (Cabanillas and Novak, 2016). Aryl hydrocarbon receptor (AhR) and sirtuin1 (SIRT1), which regulate the upregulation of barrier proteins, have attracted increasing attention in research related to skin barrier and inflammation. A study showed that the ligation of AhR activates the downstream transcription factor, OVO-like 1 (OVOL1), which then induces filaggrin expression (Lee et al., 2020). Moreover, it has been demonstrated that SIRT1, a member of the silent information regulator 2 family which catalyzes NAD^+^-dependent protein deacetylation (Bauer-Mehren et al., 2010), regulates filaggrin expression through AhR, thus indicating new roles of these markers in skin barrier function and shedding light on the molecular mechanisms that help maintaining the skin barrier and preventing inflammatory skin disease.

To investigate the potential activity of RXA on skin, we investigated the molecular mechanisms involved in the anti-AD activity of RXA and suggested the targeted potential signal pathway, using house dust mite *Dermatophagoides farinae* body (Dfb)-induced AD murine model, human keratinocytes and human skin equivalent (HSE) model.

## Materials and Methods

### Dfb-Induced AD Model

**FIGURE 1:**
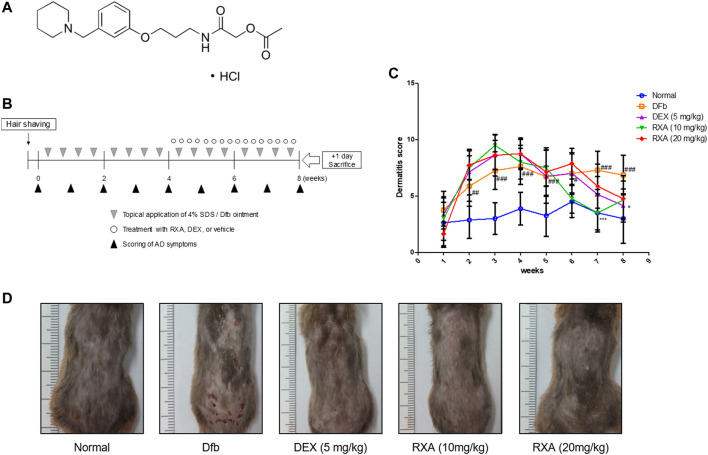
Effects of RXA on the development of Dfb-induced AD-skin symptoms in NC/Nga mice. **(A)** Chemical structure of RXA. **(B)** Schematic representation of the experiment. **(C)** Clinical features of AD-skin symptoms. **(D)** Dermatitis scores were measured once a week for 8 weeks. The dermatitis score was defined as the sum of the scores graded for each of the symptoms. The data shown represent mean ± S.D. of three independent experiments (*n* = 8). ^#^ *p* < 0.05, ^##^ *p* < 0.01, ^###^ *p* < 0.001 vs. the control group; ^*^ *p* < 0.05, and ^***^ *p* < 0.001 vs. Dfb-treated group. Dfb, *Dermatophagoides farinae* body.

Forty NC/Nga male mice (6 weeks old; 20–25 g body weight) were obtained from Daehan BioLink (Eumsung, Korea), a branch of Charles River Japan (Kanagawa, Japan) and maintained under the following conditions: temperature of 20–25°C, humidity of 40–60%, and a 12-h light/dark cycle. The mice were randomly assigned to one of the four groups (*n* = 8 per group): control group, Dfb-induced group, dexamethasone (DEX; positive control) group, and RXA (10 and 20 mg/kg, orally administered) group. The chemical structure is described in Figure 1A. To induce AD-like skin lesions, the shaved dorsal area was topically treated with 100 mg crude extract of Dfb body (Biostir-AD; Biostir, Hyogo, Japan). Mite antigen application was repeated twice a week for 8 weeks. Barrier disruption was achieved by the application of 150 μL of 4% sodium dodecyl sulfate treatment, 3 h before the application of Dfb ointment. After the first challenge of induction of AD-like symptoms for 4 weeks, the mice were treated orally with DEX [5 mg/kg dissolved in phosphate-buffered saline (PBS)] or RXA (10 and 20 mg/kg) for 4 h after Dfb treatment once a day. The concentration and administration route of RXA were determined in reference to previous study (Lee et al., 2017). The mice were sacrificed at the end of the experiment. The same volume of PBS was administered to the control group. The experimental scheme is summarized in Figure 1B. Skin tissues from the back of the mice were obtained and subjected to histological and Western blot analyses. All procedures were performed in accordance with University guidelines and approved by the Ethical Committee for Animal Care and the Use of Laboratory Animals, Korean Medicine, Sangji University (Wonju, Korea; approval no. 2017–16).

### Cytokine Assays

Dorsal skin protein extracts were suspended in PRO-PREP™ protein extraction solution (Intron Biotechnology, Inc., Seoul, Korea) and incubated for 20 min at 4°C. Debris was removed *via* micro-centrifugation 11,000 × g for 30 min at 4°C, followed by rapid freezing of the supernatant. The protein concentration was determined using Bio-Rad protein assay reagent (Bio-Rad Laboratories, Inc., Hercules, CA, United States), according to the manufacturer’s protocol. The level of interleukin (IL)-6 was measured using IL-6 DuoSet ELISA kit (R&D Systems, Inc., Minneapolis, MN, United States) according to the manufacturer’s protocol. Culture medium of human skin equivalents was collected after treatment and stored at −80°C. TNF-α, and IL-1β levels were measured in the culture supernatants using DuoSet ELISA kits (R&D Systems, Inc., Minneapolis, MN, United States) according to the manufacturer’s protocol.

### Histamine and Immunoglobulin E Assay

Blood from each mouse was collected at the end of the experiment. Serum was obtained by centrifugation at 1,700 × g for 30 min and stored at −80°C until analysis. The released histamine and IgE levels were measured using an ELISA kit, in accordance with the manufacturer’s protocol.

### Cell Culture and Sample Treatment

HaCaT keratinocytes were provided by Professor Jae-Young Um (Kyung Hee University, Republic of Korea), and were grown at 37°C in Dulbecco’s modified Eagles medium (DMEM), supplemented with 10% fetal bovine serum (FBS), penicillin (100 U/ml), and streptomycin (100 μg/ml) in a humidified atmosphere of 5% CO_2_. HaCaT keratinocytes were seeded at a density of 1 × 10^5^ cell per well, starved with 0.1% FBS media for 24 h, and treated with RXA (10, 20 and 40 μM) or Dex (0.1 μM as described in the previous study (Oh et al., 2021)) for 1 h at 37°C in humidified air with 5% CO2, and then stimulated with 10 ng/ml of TNF-α/IFN-γ at 37°C. The cells treated with dimethyl sulfoxide (DMSO) were used as control.

### AD-like Human Skin Equivalent Model

A HSE model (Neoderm®-ED) was purchased from TEGO Science, Inc. (Seoul, Korea). The HSE model contained epidermis and dermis. AD cocktail was used to induce AD-like symptoms in HSE model according to the previously described (Lee et al., 2020). In brief, an AD cocktail consisting of IL-4 (30 ng/ml), IL-13 (30 ng/ml), and TNF-α (3.5 ng/ml) with or without supplementation with RXA (40 μM) was added to the culture medium for 6 days. The culture medium was changed every 2 days.

### Statistical Analysis

The data are expressed as the mean ± standard deviation of triplicate experiments. Statistically significant differences were compared using one-way analysis of variance and Dunnett’s post hoc test. *p* < 0.05 was considered to indicate a statistically significant difference. Statistical analysis was performed using SPSS statistical analysis software (version 19.0, IBM SPSS, Armonk, NY, United States).

The detailed protocols are described in the Supplementary Materials online.

## Results

### Roxatidine Acetate Chloride Alleviated Clinical Severity of AD Skin Symptoms in Dfb-Induced AD Mice

**FIGURE 2:**
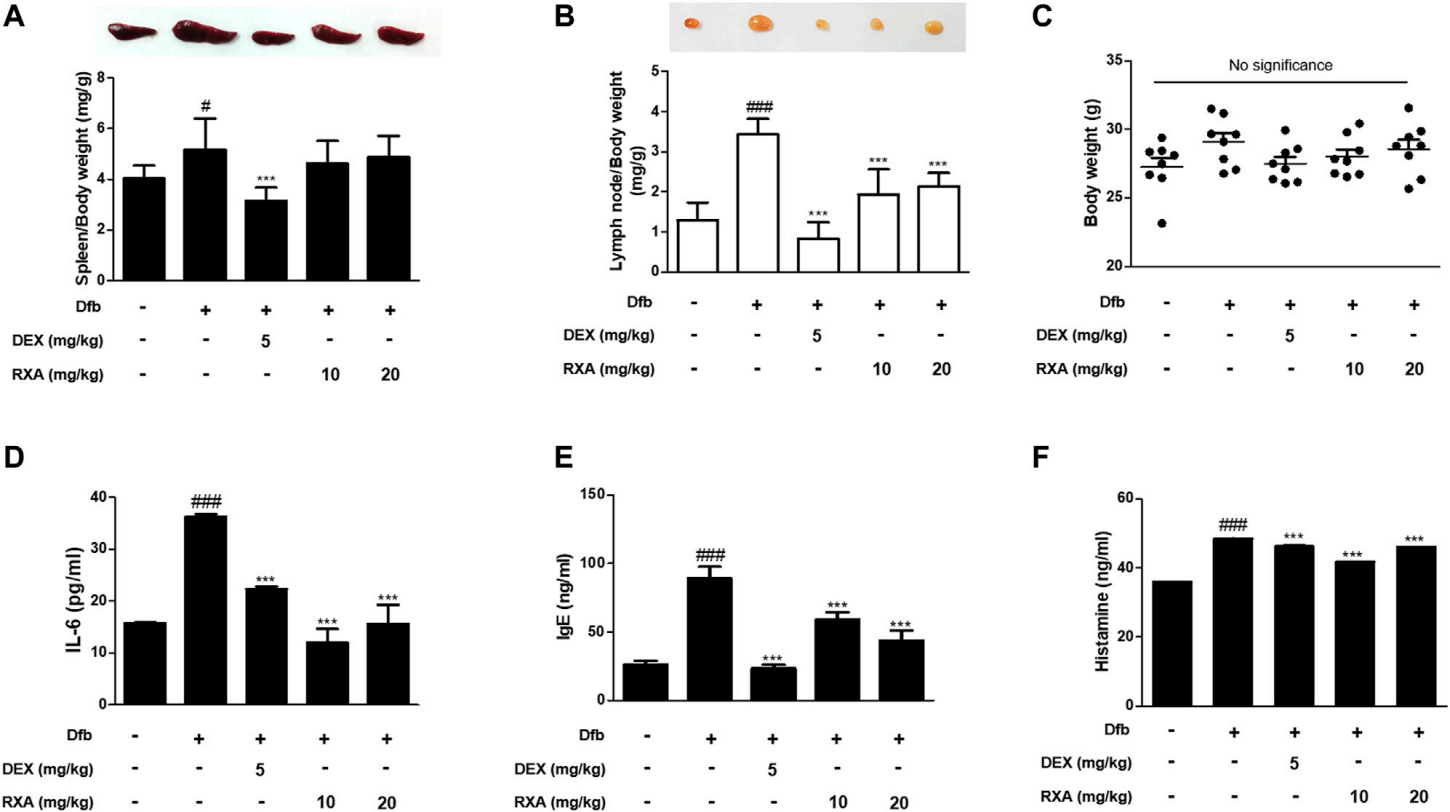
Effects of RXA on the spleen, lymph node, and the production of mediators of AD in Dfb-induced NC/Nga mice. Representative size and weight of **(A)** spleen and **(B)** lymph node were compared by photographic images. Weights of organs and whole body were measured. **(C)** Body weights at the end of 8 weeks of experimentation. The production of **(D)** pro-inflammatory cytokines, **(E)** serum IgE, and **(F)** histamine release were measured by ELISA. The data shown represent mean ± S.D. of three independent experiments (*n* = 8). ^#^ *p* < 0.05, ^###^ *p* < 0.001 vs. the control group; ^***^ *p* < 0.001 vs. Dfb-treated group. Dfb, *Dermatophagoides farinae* body.

We evaluated the effects of RXA on AD skin symptoms using an NC/Nga AD mouse model. Treatment of mice with Dfb resulted in severe AD skin symptoms, with a significant increase in dermatitis score compared with the control group. The oral administration of RXA in mice led to a noticeable reduction in dermatitis score and AD skin symptoms, which were similar to the DEX-administrated group (Figures 1C,D). Additionally, the morphological changes in the spleen and lymph node of AD mice were measured. It was observed that the spleen and lymph node of the mice in the Dfb group were significantly swollen due to inflammation, whereas those in the RXA-treated mice were smaller and lower in weight (Figures 2A,B). However, there was no significant difference in body weights between groups at the end of 8 weeks of experimentation (Figure 2C).

### Roxatidine Acetate Chloride Suppressed the Production of AD Mediators in Dfb-Induced AD Mice

The skin inflammatory response involves the release of several pro-inflammatory cytokines, including IL-6, IL-1β, and tumor necrosis factor (TNF)-α (Akram et al., 2016). Therefore, we investigated the effects of RXA on the production of AD mediators in mice. The Dfb-induced increase in IL-6 levels in the dorsal skin of AD mice was higher compared with the control group. This effect was significantly suppressed in mice that were administered RXA. The serum levels of IgE and histamine, which are the major hallmarks of AD symptoms, were also measured in AD mice serum. Results showed that administration of RXA produced significantly less serum levels of IgE and histamine in AD mice than in the Dfb group. These data indicated that RXA ameliorated AD symptoms in mice, and thus RXA may be an effective regulator of immune responses and inflammation in AD (Figures 2D–F).

### Roxatidine Acetate Chloride Normalized Histopathological Features in Dfb-Induced AD Mice Skin

**FIGURE 3:**
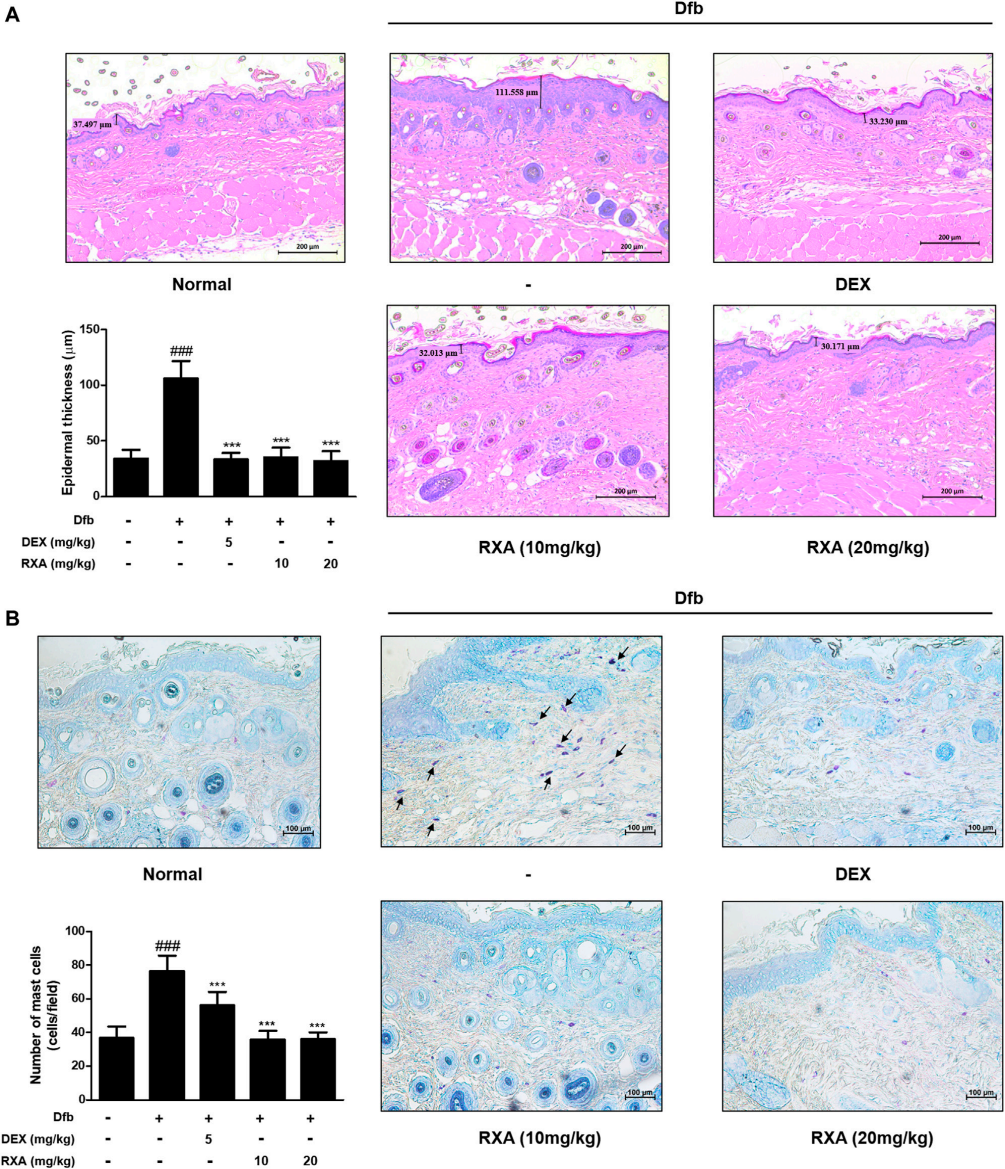
Effects of RXA on the epidermal thickness and mast cell infiltration in the dorsal skin of the Dfb-induced NC/Nga mice. **(A)** Histological features of the dorsal of NC/Nga mice. Tissues were excised, fixed in 10% formaldehyde, embedded in paraffin, and sectioned. The sections were stained with H&E (scale bar = 200 μm). Epidermal and dermal thickness in H&E stained sections were measured under a microscope. **(B)** The sections were stained with toluidine blue to identify mast cells. Blue arrows indicate stained mast cells. Mast cells were counted with a microscope (scale bar = 100 μm). The data shown represent mean ± S.D. of three independent experiments (*n* = 8). ^###^ *p* < 0.001 vs. the control group; ^***^ *p* < 0.001 vs. Dfb-treated group. Dfb, *Dermatophagoides farinae* body.

Hematoxylin and eosin (H&E) staining revealed acanthosis of the epidermis with hyperkeratosis in the Dfb group which was not observed in the control group. However, treatment with RXA significantly reduced the epidermis thickness (Figure 3A). In addition, we observed an increase in mast cell infiltration in the skin of mice in the Dfb group, which reduced after treatment with RXA (Figure 3B). These results suggested that RXA attenuates AD symptoms through reduction of mast cell infiltration following skin inflammation and prevents hyperkeratosis, hypertrophy, and thickened epidermis.

### Roxatidine Acetate Chloride Recovered Filaggrin Expression and Inhibited Expression of Adhesion Molecules in Dfb-Induced AD Mice Skin

**FIGURE 4:**
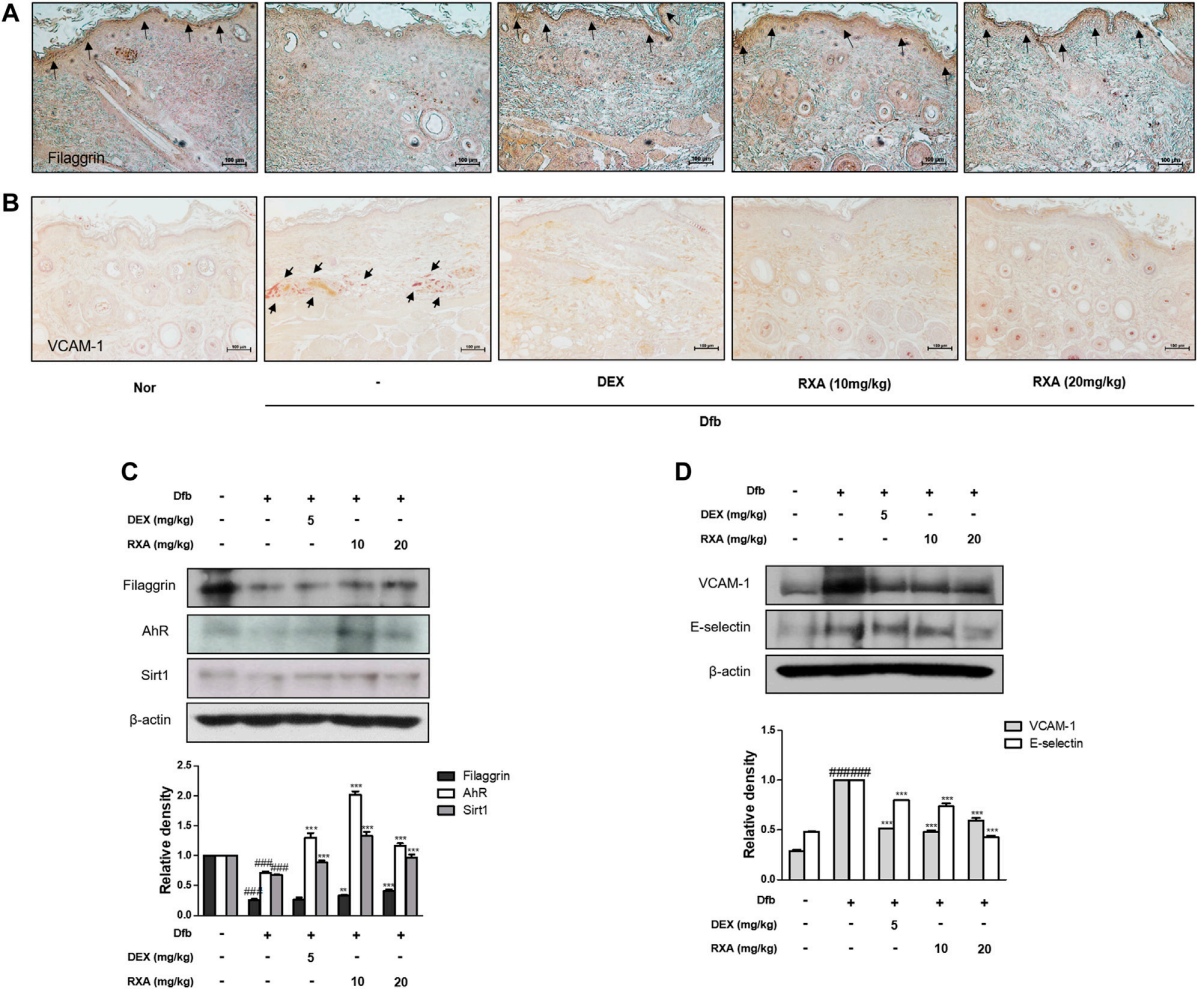
Effects of RXA on the expression of filaggrin, SIRT1, AhR, and adhesion molecules in Dfb-induced NC/Nga mice. Skin sections were stained with immunohistochemical staining. Dexamethasone (5 mg/kg, DEX) was used as the positive control. **(A)** Filaggrin in stratum corneum of epidermis and **(B)** VCAM-1 in dermis of the mouse skin were detected using specific antibodies. Black arrows indicate stained regions (scale bar = 100 μm). Total proteins were prepared and analyzed by Western blotting technique for **(C)** filaggrin, AhR, SIRT1 and **(D)** adhesion molecules, using specific antibodies. β-actin was used as the internal control. The data shown represent mean ± S.D. of three independent experiments (*n* = 8). ^###^ *p* < 0.001 vs. the control group; ^**^ *p* < 0.01, ^***^ *p* < 0.001 vs. Dfb-treated group. Dfb, *Dermatophagoides farinae* body; AhR, aryl hydrocarbon receptor; SIRT1, sirtuin1.

Using gene-disease relation analysis, we found that AD is associated with many genes, including filaggrin gene (*FLG*), *AHR*, *OVOL1*, and *SIRT1* (Supplementary Figure S1). It is reported that SIRT1 promotes the activation of AhR, in turn, enhanced AhR induces FLG expression *via* OVOL1 in normal human epidermal keratinocytes, suggesting AhR-OVOL1-FLG axis is a potent target for treatment of AD (Mulero-Navarro and Fernandez-Salguero, 2016; Tsuji et al., 2017). Based on this result, to explore whether RXA has an effect on the skin barrier function, we evaluated the expression of filaggrin. Filaggrin is a key component that is responsible for preserving the normal skin barrier function. This protein is pivotal for proper differentiation of skin cells into flat corneocytes in stratified epidermis (Egawa and Kabashima, 2018). Results showed that the expression of filaggrin was reduced after Dfb application compared with the control group. In contrast, RXA restored the protein expression of filaggrin in stratum corneum of epidermis and this effect was greater when compared with the DEX-treated group (Figures 4A,C). The transcription factor AhR in involved in accelerating epidermal terminal differentiation, and the upregulated expression of filaggrin, and this signaling is mediated via the AhR-OVOL1 axis. Moreover, filaggrin is modulated by SIRT1, which is critical for maintaining skin barrier integrity. We found that the protein levels of AhR and SIRT1 were increased by RXA in Dfb-induced mice (Figure 4C). We also investigated the effects of RXA on adhesion molecules which are involved in development of AD. The expression of vascular cell adhesion protein 1 (VCAM-1) in dermis of AD dorsal skin tissue was markedly increased upon repeated Dfb application. However, administration of RXA significantly reduced that level in AD mice skin. RXA had a similar effect on E-selectin, another adhesion molecule, in Dfb-induced mice (Figures 4B,D).

### Roxatidine Acetate Chloride Regulated the Expressions of Filaggrin, AhR, SIRT1, and OVOL1 in TNF-α/IFN-γ-Stimulated HaCaT Keratinocytes

**FIGURE 5:**
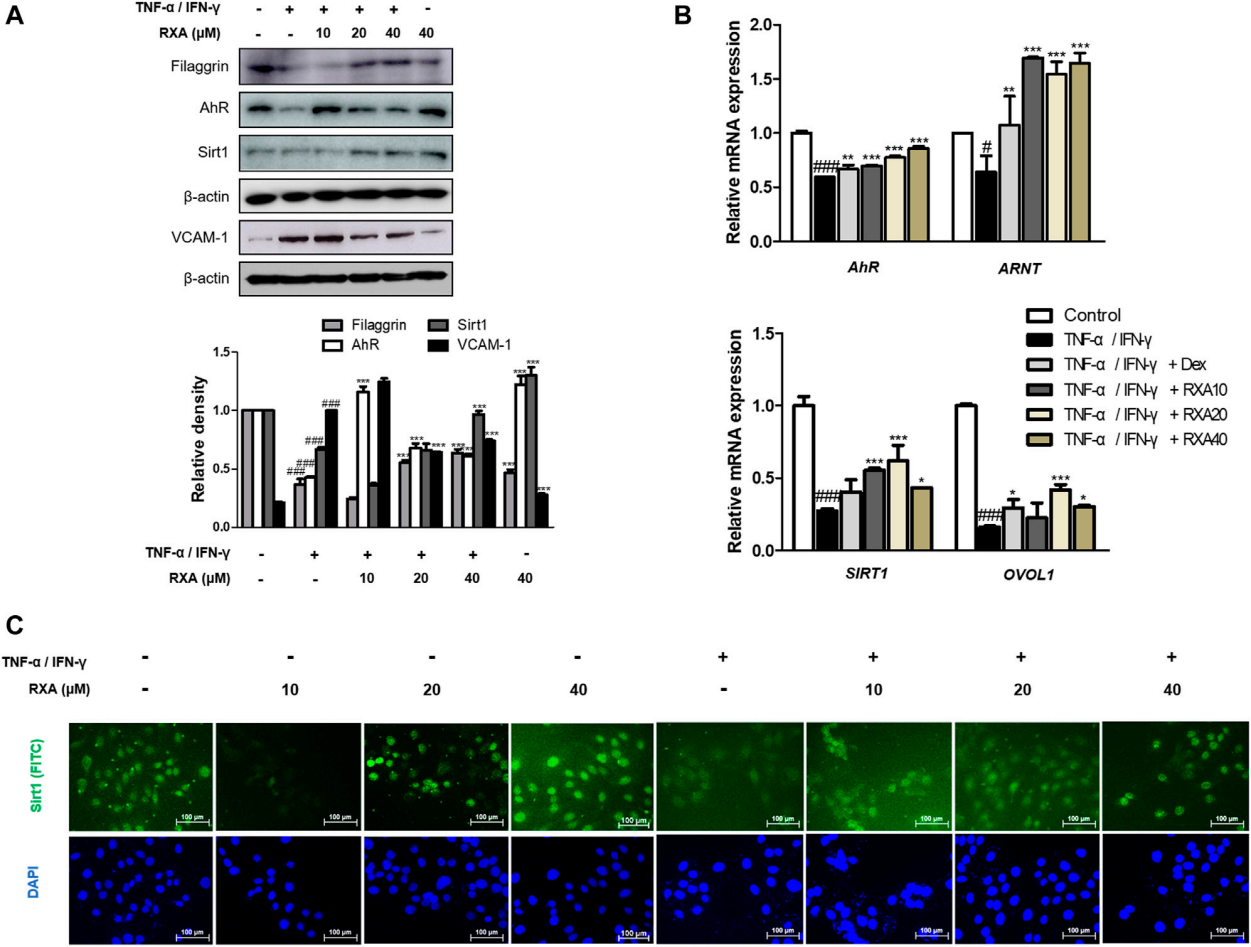
Effects of RXA on the expression of filaggrin, AhR, and SIRT1 in TNF-α/IFN-γ-stimulated HaCaT keratinocytes. HaCaT keratinocytes were pre-treated with RXA and then stimulated with TNF-α/IFN-γ. **(A)** Total proteins were prepared and analyzed by the Western blotting technique for filaggrin, AhR, SIRT1, and VCAM-1, using specific antibodies. **(B)** The mRNA levels of AhR, ARNT, SIRT1, and OVOL1 were determined by qRT-PCR. **(C)** Immunofluorescence staining of SIRT1 (green), treated with or without TNF-α/IFN-γ, in HaCaT keratinocytes. Nuclei were counterstained using DAPI (scale bar = 100 μm). The data shown represent mean ± S.D. of three independent experiments. ^#^ *p* < 0.05, ^###^ *p* < 0.001 vs. the control group; ^*^ *p* < 0.05, ^**^ *p* < 0.01, and ^***^ *p* < 0.001 vs. TNF-α/IFN-γ-stimulated group. ARNT, aryl hydrocarbon receptor nuclear translocator; OVOL1, OVO-like 1; Dex, Dexamethasone.

Based on the *in vivo* result, we examined the effect of RXA on the skin barrier-related markers in human keratinocytes. Similar to the results obtained in the AD mouse model, the reduced protein expression of not only filaggrin but also AhR and SIRT1 were recovered after treatment with RXA in TNF-α/IFN-γ-stimulated HaCaT keratinocytes. TNF-α/IFN-γ significantly induced the expression of VCAM-1, which was inhibited by RXA pre-treatment (Figure 5A). Of note, these effects were not caused by cytotoxicity since the concentrations of RXA that do not affect cell viability were selected through the cell viability assay (Supplementary Figure S2). The results showed a correlation with the results obtained for the dorsal skin of AD mice. Furthermore, we found that RXA markedly increased the mRNA levels of AhR, aryl hydrocarbon receptor nuclear translocator (ARNT, which forms a heterodimer with the AhR), OVOL1 (the downstream transcription factor of AhR), and SIRT1 compared to TNF-α/IFN-γ-stimulated group and Dex-treated group in HaCaT keratinocytes (Figure 5B). SIRT1 was mainly located in the nuclear region of the cells and decreased by TNF-α/IFN-γ stimulation in untreated HaCaT keratinocytes. However, our results show elevated levels of SIRT1 in HaCaT keratinocytes compared to TNF-α/IFN-γ-stimulated cell, irrespective of whether or not they were stimulated by TNF-α/IFN-γ (Figures 5C,D).

### Roxatidine Acetate Chloride Suppressed the Activation of NF-κB Signal Pathway in TNF-α/IFN-γ-Stimulated HaCaT Keratinocytes

**FIGURE 6:**
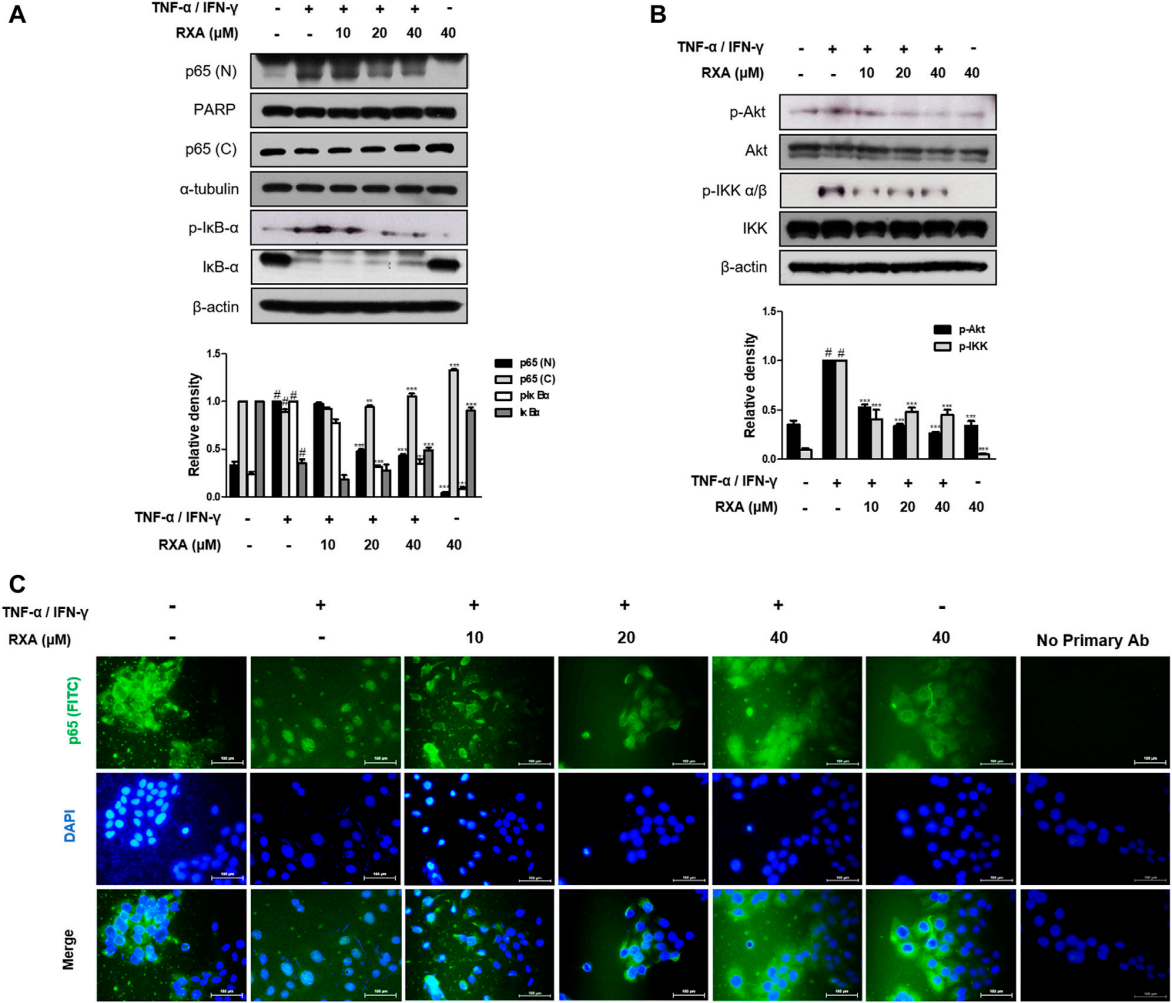
Effects of RXA on the NF-κB activation in TNF-α/IFN-γ-stimulated HaCaT keratinocytes. **(A)** Cells were pre-treated with RXA and then stimulated with TNF-α/IFN-γ. Nuclear (N) and cytosol (C) extracts were isolated, and levels of p65 in fractions were determined by Western blotting. Total proteins were prepared and Western blotted for IκB-α, **(B)** Akt, and IKK. β-actin, PARP, and α-tubulin were used as internal controls. **(C)** Immunofluorescence staining of NF-κB (green) in TNF-α/IFN-γ-stimulated HaCaT keratinocytes. Nuclei were counterstained using DAPI (scale bar = 100 μm). Secondary antibody only control was used as negative control. The data shown represent mean ± S.D. of three independent experiments. ^#^ *p* < 0.05 vs. the control group; ^**^ *p* < 0.01, ^***^ *p* < 0.001 vs. TNF-α/IFN-γ-stimulated group.

NF-κB serves as a pivotal mediator of inflammatory diseases (Liu et al., 2017), therefore, to determine whether RXA has an effect on the NF-κB signaling pathway, we performed Western blot and immunofluorescence staining. As shown in Figure 6A, RXA inhibited the TNF-α/IFN-γ-induced NF-κB p65 translocation, thus indicating that NF-κB activation is required for AD responses. Since NF-κB activation is accompanied by phosphorylation and degradation of IκBα, we evaluated whether RXA could inhibit the signaling events. As expected, the cells treated with TNF-α/IFN-γ showed phosphorylation and degradation of IκBα, which was reversed by RXA treatment. To elucidate the inhibitory effect of RXA in the upstream event of NF-κB, we investigated the Akt and IKK signaling pathways. Results showed that pre-treatment with RXA also inhibited TNF-α/IFN-γ-stimulated Akt and IKK phosphorylation (Figure 6B) in TNF-α/IFN-γ-stimulated HaCaT keratinocytes. Immunofluorescence staining of NF-κB was exclusively distributed in the cytoplasmic compartment prior to TNF-α/IFN-γ stimulation. Treatment with TNF-α/IFN-γ resulted in the translocation of NF-κB in the nucleus. This event was markedly attenuated by RXA treatment (Figure 6C). Overall, these results suggest the potential role of NF-κB in the suppression of inflammatory mediator production by RXA and indicate that the expression of filaggrin and adhesion molecules is associated with the NF-κB signaling pathway.

### Roxatidine Acetate Chloride Attenuated the Skin Inflammation in the AD-like Human Skin Equivalent Model

**FIGURE 7:**
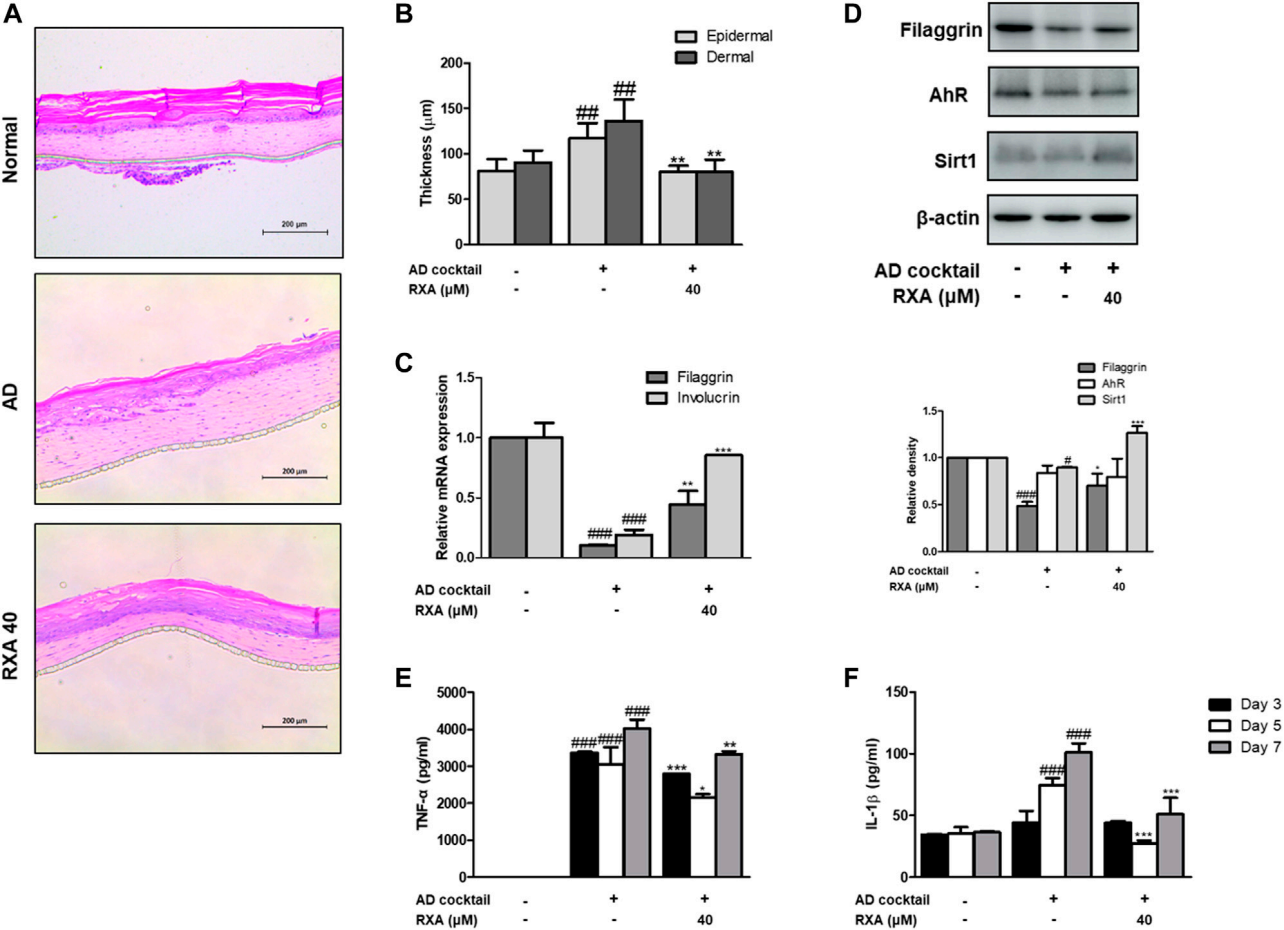
Effects of RXA on the AD-like HSE model. **(A)** Histological features of the AD-like HSE model. Tissues were excised, fixed in 10% formaldehyde, embedded in paraffin, and sectioned. The sections were stained with H&E. Blue bar indicates epidermis. **(B)** Epidermal and dermal thickness in H&E stained sections were measured under a microscope. **(C)** The mRNA levels of filaggrin and involucrin were determined by qRT-PCR. **(D)** Total proteins were prepared and analyzed by Western blotting technique for filaggrin, AhR, and SIRT1, using specific antibodies. β-actin was used as the internal control. The production of **(E)** TNF-α and **(F)** IL-1β was measured at day 3, 5, 7 by ELISA. The data shown represent mean ± S.D. of three independent experiments (*n* = 3). ^#^ *p* < 0.05, ^##^ *p* < 0.01, ^###^ *p* < 0.001 vs. the control group; ^*^ *p* < 0.05, ^**^ *p* < 0.01, and ^***^ *p* < 0.001 vs. AD cocktail-treated group.

To test the therapeutic potential of RXA on AD development, an *ex vivo* AD-like HSE model was used. We found that RXA treatment significantly reduced epidermal and dermal thickness (Figures 7A,B). AD cocktail-induced decreases of filaggrin and involucrin were recovered in the AD-like HSE model (Figure 7C). Moreover, the protein levels of filaggrin and Sirt1 in AD-like HSE model were increased by RXA treatment compared with those in AD cocktail-treated group, though, the expression of AhR did not show any difference between groups (Figure 7D). Increased levels of the TNF-α and IL-β induced by AD cocktail were suppressed by RXA treatment in AD-like HSE model (Figures 7E,F). RXA treatment significantly decreased the TNF-α level produced from stimulated HSE tissue despite of the TNF-α portion contained in AD cocktail (Figure 7E).

## Discussion

AD is a chronic and relapsing inflammatory skin disease, which is related with alterations in skin barrier factors and imbalance in the immune system. Taking into consideration the inside-outside theory, the abnormal immune response in AD has been thought of as the main mechanism for inflammation and cause of increased skin permeability. However, the skin barrier dysfunction in AD has proved to exhibit an important role in the pathogenesis of AD (Zaniboni et al., 2016). For better understanding of AD with a complex pathogenesis, it is necessary to approach from a more diverse and differentiated strategy. One of them is to find out alternate therapeutic uses of existing drugs. The histamine H_2_ receptor antagonist, RXA, suppresses gastric acid secretion by inhibiting the histamine H_2_ receptors located in gastric parietal cells (Nakamura et al., 2012). It has been long known that histamine may play a key role in the pathogenesis of AD. Despite the fact that anti-histamines have used in the therapeutic medication of AD patients suffering from itch to alleviate the symptoms, the benefits of anti-histamine treatment were not supported by substantial evidence (Buddenkotte et al., 2010). Recently, Ashida *et al.* suggested that both the histamine H1 and histamine H2 receptors contribute to the maintenance of skin barrier function (Ashida et al., 2001), and histamine H4 receptor antagonists displayed anti-pruritic and anti-inflammatory effects not only murine models but also in clinical studies (Schaper-Gerhardt et al., 2020). These findings imply that the histamine receptors and their antagonists may also be interesting therapeutic targets in AD patients. In this study, we examined the anti-AD effect of RXA and suggested new insights into the underlying mechanisms of RXA, using an AD murine model, human keratinocytes, and AD-like HSE model.

The epidermal differentiation marker, Filaggrin contributes to generation of the natural moisturizing factors and may be important in maintaining the immune defense mechanism and integrity of the skin (Sandilands et al., 2009). There has been a growing body of evidence to suggest that mutations in the *FLG* is a strong risk factor for AD and allergic diseases (Esparza-Gordillo et al., 2015). We also observed that Dfb or TNF-α/IFN-γ decreased the expression of filaggrin and transcription factor AhR. However, treatment with RXA restored the expression of these components in both AD mouse model and human keratinocytes, and the expression of filaggrin was significantly enhanced in the AD cocktail-induced AD-like HSE model, thus suggesting that RXA has a promising effect in enhancing the skin barrier function through the modulation of filaggrin and AhR. Meanwhile, the genetic deletion of SIRT1 in normal human keratinocytes and mouse skin downregulates *FLG* with AD-like skin lesions, indicating that the deacetylase activity of SIRT1 is required for the regulation of *FLG* expression. In addition, SIRT1 regulates *FLG* expression through AhR, suggesting an indirect mechanism in *FLG* regulation by the SIRT1/AhR pathway with its partner, ARNT, which induces the expression of *FLG* and other epidermal structural proteins (Robertson et al., 2012). In line with the previous studies on the therapeutic effect of AhR activation (van den Bogaard et al., 2013), we observed reinforcing effects of RXA on SIRT1, AhR, and filaggrin in both of Dfb-induced AD animal skin, TNF-α/IFN-γ-stimulated HaCaT keratinocytes, and AD-like HSE model.

Studies have shown that the induction of adhesion molecules can be blocked by neutralizing antibodies to pro-inflammatory cytokines, implying that these cytokines are involved in the expression of adhesion molecules (Detmar et al., 1992). Furthermore, it has been reported that inflammatory cytokines play an important role in regulating epidermal homeostasis, innate barrier function, and filaggrin down-regulation in keratinocytes (Noh et al., 2010; Son et al., 2014). Therefore, modulating the expression of filaggrin and adhesion molecules is a promising strategy to treat inflammatory skin disorders. We showed that RXA reversed the expression of filaggrin and VCAM-1 in both of Dfb-induced AD animal skin and TNF-α/IFN-γ-stimulated HaCaT keratinocytes, suggesting that the suppression of IL-6 level by RXA is implicated in its alleviatory effect against AD. The expression of VCAM-1 in keratinocytes in this study supports the reports that other types of cells than endothelial cell may express VCAM-1, including bronchial epithelial cells (Atsuta et al., 1997), renal tubular epithelial cells (Wuthrich et al., 1993), tonsil epithelial cells (Uccini et al., 1993) and fibrohistiocytic cells (Kim et al., 2002) in the setting of inflammatory environment. Moreover, we obtained the results that RXA significantly inhibited IKK phosphorylation, IκBα degradation, and p65 translocation in this *in vitro* model. The results suggested that RXA modulates the pro-inflammatory mediators and adhesion molecules by suppressing the signaling cascades leading to NF-κB activation. Notably, it has been reported that SIRT1 activators suppress inflammatory responses through promotion of p65 deacetylation and inhibition of NF-κB activity, thus indicating that increasing SIRT1-mediated NF-κB deacetylation is a novel approach for the development of a new therapeutic anti-inflammatory agents (Liu et al., 2017). Furthermore, AhR may interact with the p65-subunit of the NF-κB transcription factors. However, AhR-signaling may be a weak activator of p65-signaling that suppresses p65-activity (van den Bogaard et al., 2013). It is thus necessary to investigate about the crosstalk between them. In present *in vivo* study, 10 mg/kg of RXA administration upregulated the expression of Sirt1 and AhR compared to 20 mg/kg of RXA group (Figure 4C). In line with this, dermatitis scores (Figure 1C), dorsal IL-6 production (Figure 2D), and serum histamine level (Figure 2F) were more lowered by treatment with 10 mg/kg of RXA than 20 mg/kg of RXA, indicating RXA may be advantageous to manage the skin inflammation. These results are in agreement with previous studies that SIRT1/AhR activation leads to suppression of inflammatory response (Purushotham et al., 2009; Crunkhorn, 2018). However, it is reported that even low concentration of roxatidine acetate caused adverse effect such as skin rash in patients with ulcers (Inoue, 1988), thus further research is needed with regard to the suitable concentration of RXA on skin, and its possible use in clinic.

Besides, AhR is a chemical sensor that is expressed abundantly in the epidermal keratinocytes. Oxidative AhR ligands induce the production of reactive oxygen species (ROS), while anti-oxidant AhR ligands inhibit ROS generation via activation of nuclear factor erythroid 2-related factor 2 (Nrf2), which is the master switch for anti-oxidative signaling (Furue et al., 2018). Interestingly, some AhR agonists, including various phytochemicals, work as antioxidants via AhR-Nrf2 activation. Considering that oxidative stress is increased in inflammatory skin conditions, anti-oxidative AhR agonists are particularly promising in drug development for AD in which inflammation, barrier disruption, and oxidative stress have a combination effect. Additionally, anti-oxidative AhR ligands reduce the production of pro-inflammatory cytokines from keratinocytes and restore the impaired epidermal barrier function, in association with *FLG* upregulation (Doi et al., 2014; Furue et al., 2018). Using this approach, it is necessary to identify the relationship between antioxidant and AD. For future studies, we will be examining the relationship between the Nrf2-related marker and AD.

In conclusion, RXA demonstrated its anti-AD activities through the activation of filaggrin/AhR/SIRT1 and suppression of NF-κB signaling. These results suggest that RXA can be used as an anti-AD agent with promising therapeutic potential, based on its anti-inflammatory and anti-oxidant activities. Given that Roxatidine’s safety has been ensured in clinical fields, the adaptation to AD may provide good strategies and insights on the new potential of Roxatidine.

## Data Availability

The original contributions presented in the study are included in the article/Supplementary Material, further inquiries can be directed to the corresponding authors.
