# Supplementary material for: New Potential of Roxatidine Acetate Hydrochloride on Atopic Dermatitis Mouse Model, Human Keratinocytes, and Human Skin Equivalent Model
Source: Front Pharmacol. 2021 Dec 24;12:797086. doi: 10.3389/fphar.2021.797086 (PMC8740129; doi:10.3389/fphar.2021.797086)
Supplement: Supplementary file 1 [file DataSheet1.docx]

**Supplementary Material**


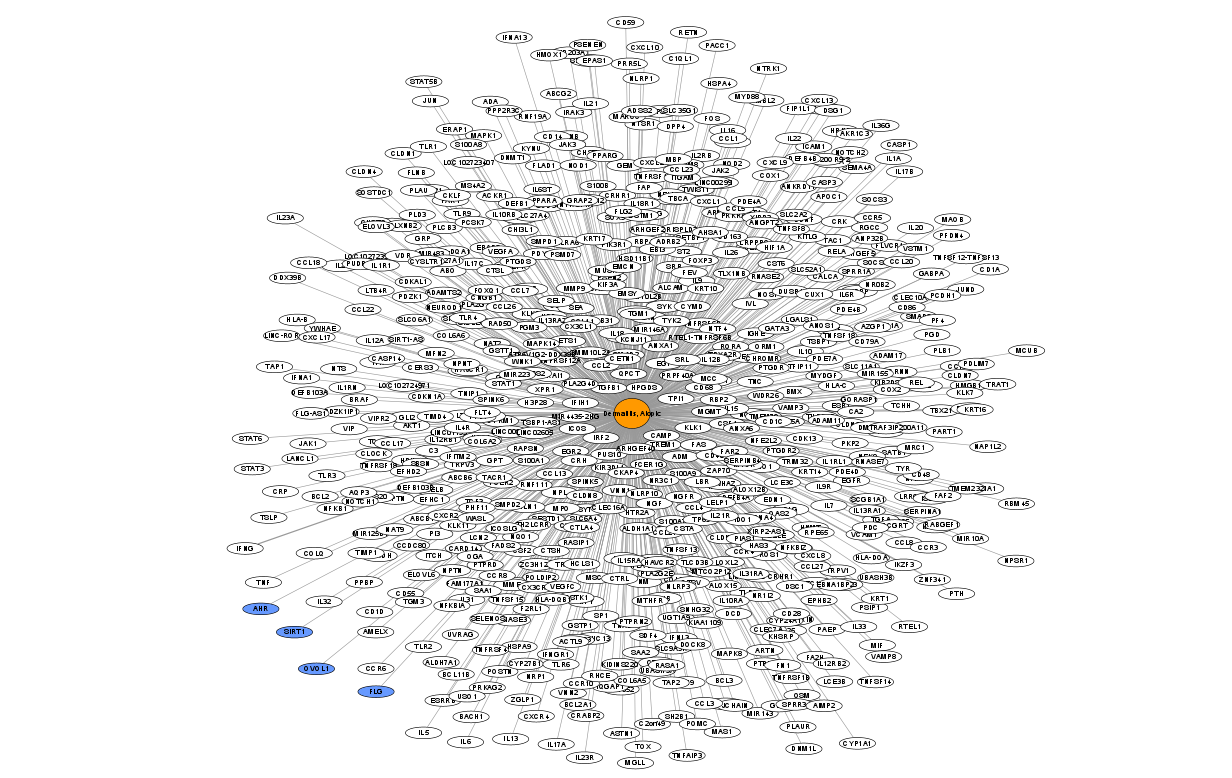


Supplementary figure 1. Gene-disease (AD) network was analyzed using DisGeNET plugin within Cytoscape. We ran the tool under the option ‘Dermatitis, Atopic’ for source node, and the correlated genes (751 genes) are presented by target node in network analysis. The AhR, SIRT1, OVOL1, and FLG genes are highlighted in blue.


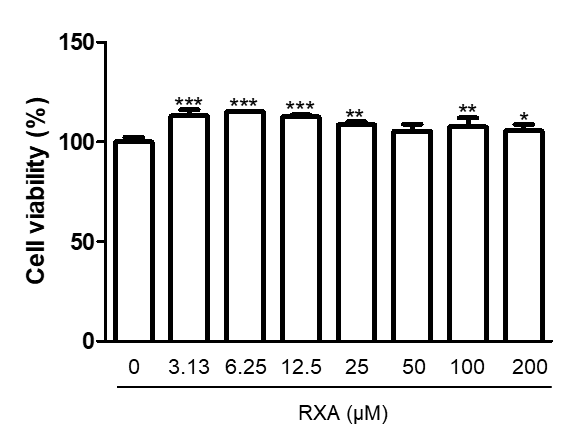


Supplementary figure 2. HaCaT keratinocytes were incubated with the indicated doses of RXA for 24 h, and cell viability was evaluated by MTT assay. The data represent mean ± S.D. of three independent experiments. ^*^ p < 0.05, ^**^p < 0.01, ^***^p < 0.001 vs. non-treated group.

**Materials and Methods**

**Chemicals and reagents**

Roxatidine acetate hydrochloride (R0178, > 98.0%) was purchased from Tokyo Chemical Industry Co., Ltd. (Chuo-ku, Tokyo, Japan). MTT, DMSO, and all other chemicals were purchased from Sigma; EMD Millipore (Billerica, MA, USA). Recombinant human TNF-α and IFN-γ were purchased from Bio-Techne Ltd. (Abingdon, OX, UK). DMEM, FBS, penicillin, and streptomycin were obtained from Life Technologies Inc. (Grand Island, NY, USA). Primary antibodies against p-IKK α/β, NF-κB p65, p-Akt, and ICMA-1 were obtained from Cell Signaling Technology, Inc. (Danvers, MA, USA). Primary antibodies for IKK α/β, p-IκB-α, IκB-α, Akt1/2/3, PARP, α-tubulin, Filaggrin, VCAM-1, E-selectin, and β-actin were purchased from Santa Cruz Biotechnology, Inc. (Dallas, TX, USA). Horseradish peroxidase-conjugated secondary antibodies were purchased from Jackson ImmunoResearch laboratories, Inc. (West Grove, PA, USA). The histamine enzyme-linked immunosorbent assay (ELISA) kit was obtained from Enzo life Sciences, Inc. (Farmingdale, NY, USA). The ELISA kits for TNF-α, IL-6, IL-1β were obtained from R&D Systems, Inc. (Minneapolis, MN, USA).

**Scoring of dermatitis severity**

Clinical dermatitis severity was measured according to the scoring system as follows. The development of skin symptoms; erythema/hemorrhage, scarring/dryness, edema, and excoriation/erosion was scored as follows: 0, none; 1, mild (< 20%); 2, moderate (20-60%); and 3, severe (> 60%). The sum of the individual scores was used as the dermatitis score. The severity of dermatitis was evaluated on the first and last days of the experiment.

**Histopathological and Immunohistochemical analysis**

At the end of the study period, the dorsal skin of mice was obtained. The samples were fixed in 10% buffered formalin, embedded in paraffin, divided into 4 μm thick sections, and stained with H&E and toluidine blue to detect epidermal thickness and inflammatory cells. For immunohistochemical staining, a portion of the skin samples from the back of the mice in each group was fixed in 10% formalin. After paraffin embedding, sections were cut and the slides were deparaffinized by xylene, rehydrated in ethanol, and rehydrated with water. Endogenous peroxidase activity was blocked using 0.6% H_2_O_2_ in 50% MeOH, and the slides were then treated with 0.3 Triton in PBS for permeabilization and pro-blocked with 10% normal goat serum (NGS) for 1 h, followed by overnight incubation with a specific antibody at 4 °C. Next, the sections were washed and incubated with horseradish peroxidase-conjugated secondary antibodies for 1 h at 20 °C. The activity was visualized with 3,3'-Diaminobenzidine chromogen and counterstained with H&E. Pathological changes of all the stained skin sections were observed using a DM IL LED microscope (Leica, Wetzlar, Germany) and photographed using a DFC295 (Leica). Digital images were taken from each slide (two per group) and measured using Leica Application Suite (Leica).

**Western blot analysis**

Segments of cells, liver tissue, or dorsal tissue were suspended in PRO-PREP™ protein extraction solution (Intron Biotechnology, Inc., Seoul, Korea) and incubated for 20 min at 4 °C. Cell debris was removed via micro-centrifugation 11,000× g for 30 min at 4 °C, followed by rapid freezing of the supernatant. The protein concentration was determined using Bio-Rad protein assay reagent (Bio-Rad Laboratories, Inc., Hercules, CA, USA), according to the manufacturer’s protocol. Cellular proteins from the treated and untreated cell extracts (10-30 μL) were electroblotted onto a polyvinylidene fluoride membrane, following separation via 8-12% SDS-PAGE. The membrane was incubated for 1 h with blocking solution (5% skim milk) at 20 °C, followed by overnight incubation with the primary antibodies (1:1,000) at 4 °C. The blots were washed thrice with Tween 20/Tris-buffered saline (T/TBS) and incubated with horseradish peroxidase-conjugated secondary antibody (1:2,000) for 2 h at 20 °C. The blots were washed thrice with T/TBS, and then developed via enhanced chemiluminescence (GE Healthcare Life Sciences, Chalfont, UK). Densitometric analysis was performed using Bio-Rad Quantity One software version 4.3.0 (Bio-Rad Laboratories, Inc., Hercules, CA, USA).

**Immunofluorescence staining**

Cells were seeded in a chamber at 1×10^5^ cells/ml, fixed with 100% methanol for 30 min at 20 °C, and blocked in 10% NGS in 0.3% Triton-X100 (Sigma). The samples were then incubated with SIRT1 or NF-κB p65 primary antibody overnight at 4 °C. After washing, the samples were incubated with the secondary antibody with Alexa-Fluor 488-conjugated goat anti-rabbit IgG (Invitrogen). Coverslips were mounted on to the glass slides and the images were captured on a confocal laser-scanning fluorescence microscope Leica TCS SP5 (LAS AF) suite (Leica Microsystems).

**Gene-disease network analyses**

The gene-disease network of AD was analyzed by using DisGeNET, which is a Cytoscape plugin to query and analyze human gene-disease networks on the basis of various sources, human gene-disease associations (GDAs) and variant-disease associations (VDAs) from various repositories including Mendelian, complex and environmental diseases as described by Bauer-Mehren ([Bauer-Mehren et al., 2010](#_ENREF_1)).

**References**

Bauer-Mehren, A., Rautschka, M., Sanz, F., & Furlong, L. I. (2010). DisGeNET: a Cytoscape plugin to visualize, integrate, search and analyze gene-disease networks. *Bioinformatics,* 26(22), 2924-2926. doi: 10.1093/bioinformatics/btq538
